# Climate-driven mosquito-borne viral suitability index: measuring risk transmission of dengue, chikungunya and Zika in Mexico

**DOI:** 10.1186/s12942-022-00317-0

**Published:** 2022-10-27

**Authors:** Constantino Carreto, Roxana Gutiérrez-Romero, Tania Rodríguez

**Affiliations:** 1 El Colegio de México (COLMEX), Carretera Picacho-Ajusco 20, Tlalpan, 14110 Mexico City, Mexico; 2 Queen Mary University of London (QMUL), Mile End Campus, Bancroft Building, 4th Floor, London, E1 4NS UK; 3 Institute of Geography, Universidad Nacional Autónoma de México (UNAM), Investigación Científica, Ciudad Universitaria, C.U., Coyoacán, 04510 Mexico City, Mexico

**Keywords:** Climate change, Health, Dengue, Zika, Chikungunya, Index P, Mosquito-borne, Vector-born-diseases, Mexico

## Abstract

**Background:**

Climate variability influences the population dynamics of the *Aedes aegypti* mosquito that transmits the viruses that cause dengue, chikungunya and Zika. In recent years these diseases have grown considerably. Dengue is now the fastest-growing mosquito-transmitted disease worldwide, putting 40 per cent of the global population at risk. With no effective antiviral treatments or vaccines widely available, controlling mosquito population remains one of the most effective ways to prevent epidemics. This paper analyses the temporal and spatial dynamics of dengue in Mexico during 2000–2020 and that of chikungunya and Zika since they first appeared in the country in 2014 and 2015, respectively. This study aims to evaluate how seasonal climatological variability affects the potential risk of transmission of these mosquito-borne diseases. Mexico is among the world’s most endemic countries in terms of dengue. Given its high incidence of other mosquito-borne diseases and its size and wide range of climates, it is a good case study.

**Methods:**

We estimate the recently proposed mosquito-borne viral suitability index P, which measures the transmission potential of mosquito-borne pathogens. This index mathematically models how humidity, temperature and precipitation affect the number of new infections generated by a single infected adult female mosquito in a host population. We estimate this suitability index across all Mexico, at small-area level, on a daily basis during 2000–2020.

**Results:**

We find that the index P predicted risk transmission is strongly correlated with the areas and seasons with a high incidence of dengue within the country. This correlation is also high enough for chikungunya and Zika in Mexico. We also show the index P is sensitive to seasonal climatological variability, including extreme weather shocks.

**Conclusions:**

The paper shows the dynamics of dengue, chikungunya and Zika in Mexico are strongly associated with seasonal climatological variability and the index P. This potential risk of transmission index, therefore, is a valuable tool for surveillance for mosquito-borne diseases, particularly in settings with varied climates and limited entomological capacity.

**Supplementary Information:**

The online version contains supplementary material available at 10.1186/s12942-022-00317-0.

## Introduction

Climate change and extreme weather events have imposed a significant threat to the growth and spread of climate-related diseases [1]. A clear example of this threat is mosquito-borne diseases which remain among the most important global health challenges [2]. For thousands of years, humans have cohabited with the *Anopheles* mosquitoes responsible for transmitting the parasite that causes malaria, and the *Aedes* mosquito, carrier of many arboviral diseases including dengue, and newly emerged viruses such as chikungunya and Zika. Because the *Aedes* mosquitoes live near and prefer to feed on the blood of humans, they are more likely to spread diseases, dengue being the most common. Approximately 2.5–4 billion people, 40–60% of the world’s population, live in areas at risk of dengue [3]. Some studies estimate that the number of dengue cases increased from nearly 23 million in 1990 to 105 million in 2017. Other studies suggest that these figures are conservative, and the real number of current infections is up to 390 million [4]. About one in four people infected with dengue get sick. Dengue symptoms are flu-like. The illness might progress to dengue haemorrhagic fever that manifests with vomiting, diarrhoea, and uncontrolled bleeding that might lead to system failure and can be fatal. During 1990–2017, the number of annual deaths caused by dengue increased from nearly 17,000 to 40,467 [5].

The rapid expansion of dengue in recent years and other mosquito-borne diseases has been attributed to various complex interactions of social, economic and ecological factors, but remain strongly influenced by climatic conditions such as changes in rainfall, humidity and temperature [4]. Warmer temperatures and humidity improve the chances of larval development, adult mosquito emergence rate, lifespan and increase the chances of virus transmission. More rainfall boosts the number of breeding sites, whilst less rainfall can also increase mosquito population dynamics if people store water in containers, which serve as mosquito breeding sites [1].

Over the last 50 years, dengue has undergone a geographic expansion and increased the number of infections in Latin America. This disease has remained the region’s most important arthropod-borne viral infection due to accelerated urban population growth and climate change [6–8]. Mexico is a clear example of the expansion of dengue, where the disease has become one of the most severe public health threats amid the ongoing COVID-19 pandemic. In the country, the incidence rate of uncomplicated dengue cases per 100,000 population increased from 1.72 in 2000 to 14.12 in 2011, with all ages affected but peaks in the age range of 10–20 [9]. By 2014, Mexico was the fourth most endemic country worldwide in confirmed dengue cases, behind Indonesia, Vietnam and Brazil [10, 11]. Chikungunya and Zika are also locally transmitted in Latin America, mainly by the bite of infected *Aedes aegypti* [12]. The first local transmission of chikungunya in the region was detected in 2013, the same year as Zika is suspected to have arrived on the continent according to phylogeographic analysis [13]. These diseases are of the same family and share a vector with dengue. Thus, changes in mosquito population that favour dengue might also favour chikungunya and Zika [14]. Yet, the outbreak dynamics of these diseases do not need to conform to the same seasonal pattern [15]. Although chikungunya and Zika rarely cause death their symptoms can be debilitating, including fever, joint and muscle pain. Crucially, if Zika is transmitted to pregnant women the virus can be passed to the foetus with irreversible health consequences including microcephaly [16].

The health risks are complex because there are no treatments for either of the three arboviruses discussed here. There are no approved vaccines for Zika and chikungunya yet. Although there is a vaccine to prevent dengue, licensed in December 2015, it has been approved in just 20 countries for people aged 9–45 that have prior laboratory-confirmed dengue infection [17]. Thus, the only long-term protective strategy is to control the mosquito population and have adequate surveillance tools to identify areas and periods at risk of mosquito-borne pathogens transmission. The local risks of transmission depend on a complex interplay between changes in local climatic conditions [18], population movements [19, 20], households’ socio-economic characteristics [11], population density [21], and various other factors that increase opportunities for mosquitoes to breed [22, 23]. However, many developing countries lack the capacity to collect such a wide range of entomological, epidemiological, socio-economic data systematically across the whole territory. Releasing regular data on the incidence of these diseases helps to monitor risks. However trends of incidence of arboviral diseases do not necessarily highlight accurately where future outbreaks might emerge nor whether the trends of chikungunya or Zika will follow the same trends of dengue [15]. Thus, there is a need for better surveillance tools with the capacity to promptly measure the risk of transmission of mosquito-borne pathogens.

In this paper we contribute to the literature in three ways. Our first contribution is to estimate the recently proposed mosquito-borne viral suitability index, known as index P, for Mexico during 2000–2020. Index P estimates the likely average number of new infections generated by a single adult female mosquito on a susceptible host population [17]. We argue that the index P can complement existing surveillance systems to promptly predict the amplitude of mosquito-borne viral transmission risk. A key advantage of the index P is that its estimation depends only on local humidity, temperature and precipitation data, all of which are readily available for most settings, at a small-area level and on a daily basis. We present the index P on a *daily* basis to illustrate how sensitive the index is to climatic fluctuations. We present this analysis during 2000–2020 for three in-depth case studies: Acapulco, Cancún and Mexico City. On the one hand, Acapulco and Cancún are coastal cities that have markedly different climate seasonal patterns, are exposed to frequent hurricanes, and high levels of population mobility. As expected, the index P reveals markedly different seasonal patterns of mosquito-borne disease risk transmission for these cities. On the other hand, Mexico City, the second most populated city in Latin America, has always experienced very low levels of mosquito-borne diseases given its climate and high altitude which reduces the vector potential as it is harder for mosquitoes to breed. As expected, the index P for this case predicts a low risk of transmission [24].

Our second contribution is to estimate the index P for the entire Mexican territory during 2000–2020 and present detailed graphical analysis of when the index P peaks regionally, focusing on 2010–2020. Although there are several alternative mosquito-borne viral suitability indices, these indices depend on information not always available or measured regularly such as on deforestation, human mobility [18, 19], urbanisation [20], mosquito population, number of female mosquitoes per human [21, 22] and water management practices [10]. Our analysis helps us to illustrate the feasibility of the estimation of the index P for a country as vast as Mexico over a prolonged period.

The index P has been estimated for a handful of other contexts and shown to correlate well with a few mosquito-borne diseases during limited period analyses. For instance, that is the case for Brazil for dengue during 2007–2012 [17], Israel for West Nile virus during 2016–2018 [23], and the Dominican Republic for dengue, chikungunya and Zika during 2012–2018 [13]. Our third contribution is to estimate the correlation between the index P and dengue across all 2469 municipalities in Mexico during 2010–2020, and with chikungunya and Zika since they first were reported in the country in 2014 and 2015, respectively. Mexico has a high degree of climate diversity, coastal areas exposed to frequent hurricanes, a high level of domestic and international population movements, and high levels of inequality and poverty. To provide a view of such diversity, we also present nine case studies across Mexico, in the north (Ciudad Mante, Mexicali, Monterrey), centre (Mexico City), southeast (Campeche, Tuxtla Gutiérrez), and coasts (Acapulco, Cancún, Coatzacoalcos). Overall, our analysis contributes to understanding the strength of correlation between the index P and mosquito-borne diseases, and the value of the index P for entomological surveillance and disease prevention.

## Setting

**Fig. 1:**
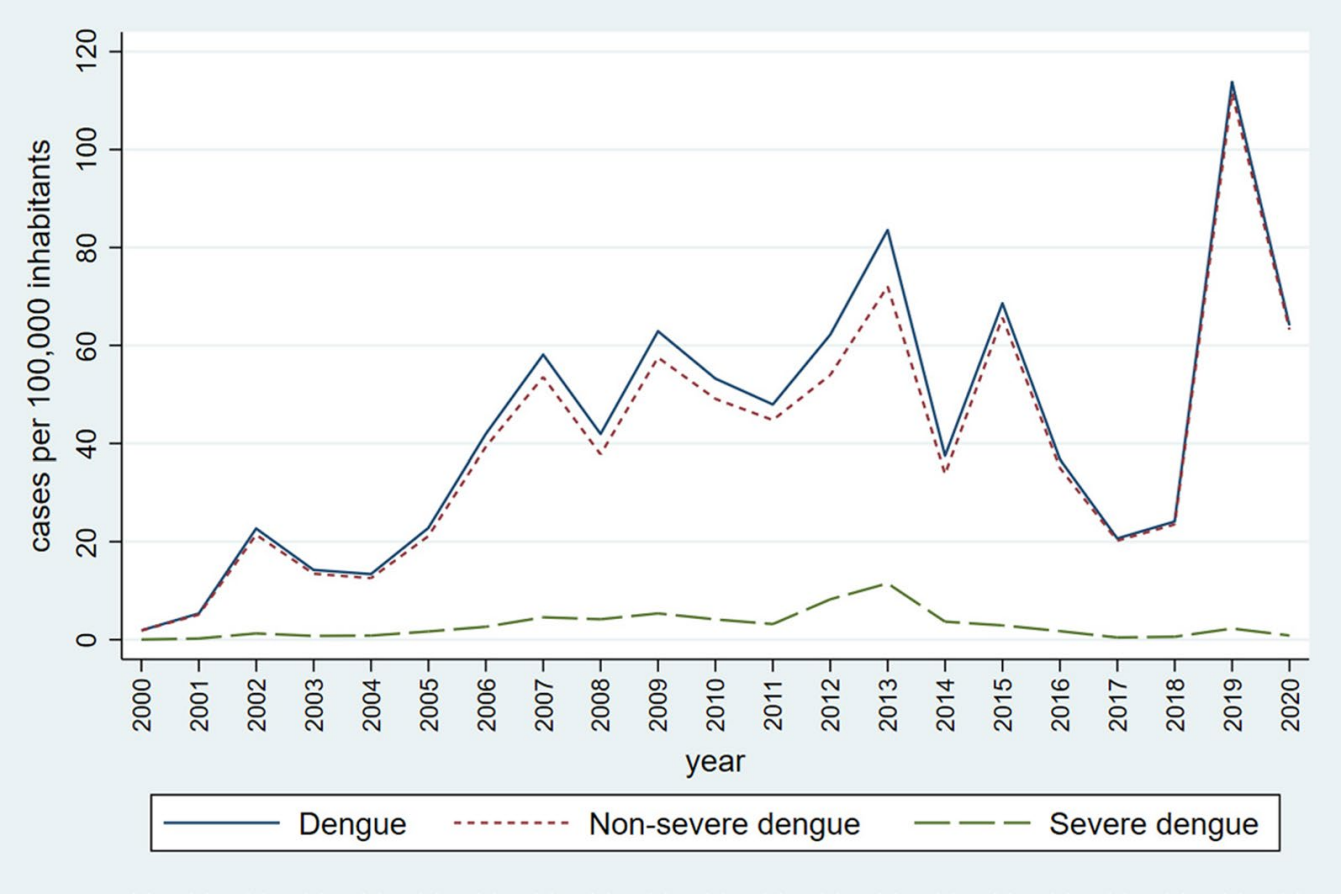
Annual dengue incidence rates during 2000–2020

**Fig. 2:**
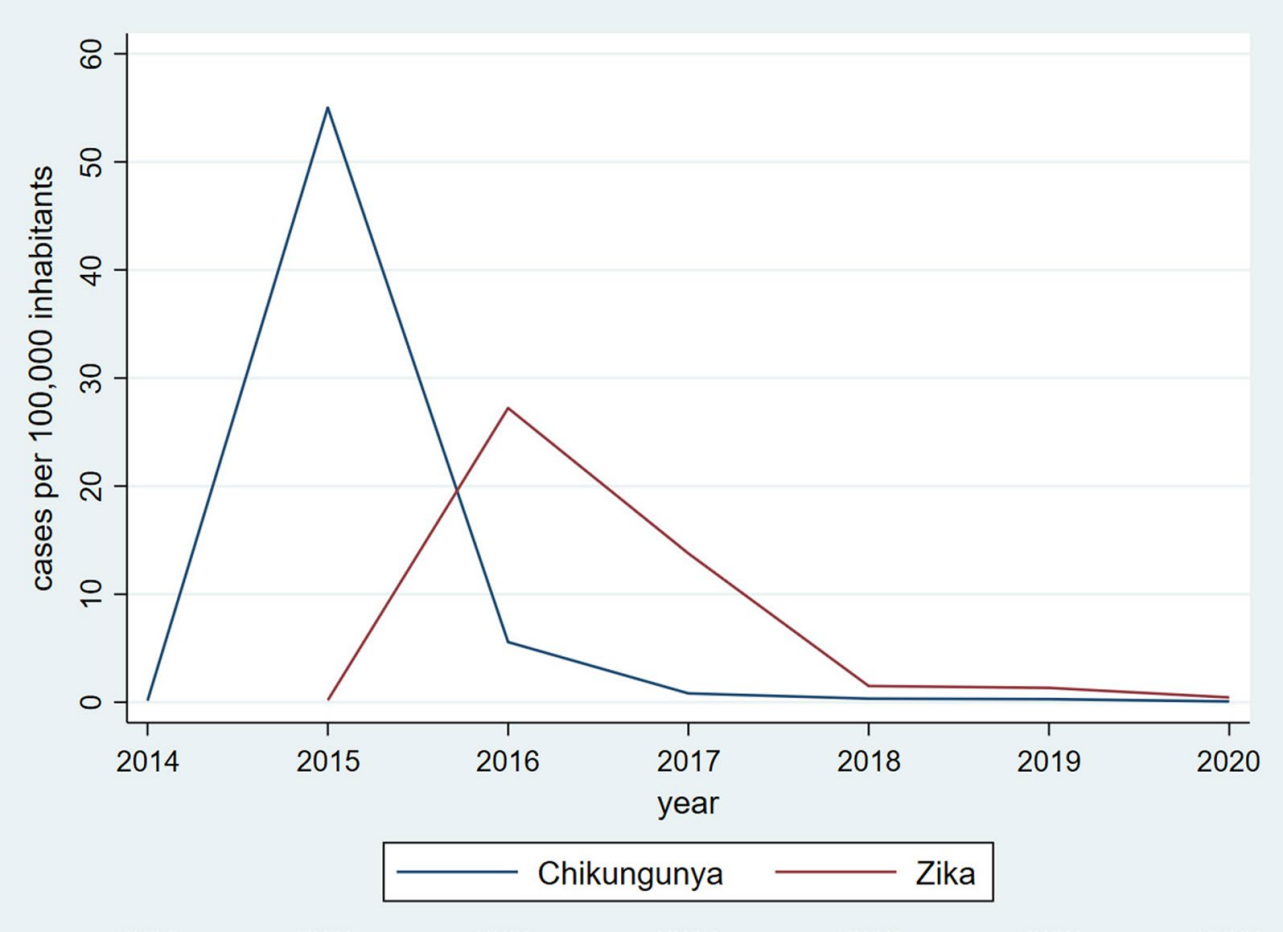
Annual chikungunya and Zika incidence rates during 2014–2020

Over the last two decades, Mexico witnessed a sharp increase in severe and non-severe dengue incidence rates. The overall dengue rate per 100,000 population increased from 1.89 in 2000 to 64.07 in 2020, experiencing clear ups and downs and sharp peaks in 2007, 2009, 2013, 2015 and 2019 (Fig. 1). In contrast, chikungunya and Zika reached the highest incidence a year after their introduction into the country in 2014 and 2015, respectively, and ever since have rapidly declined (Fig. 2).

**Fig. 3:**
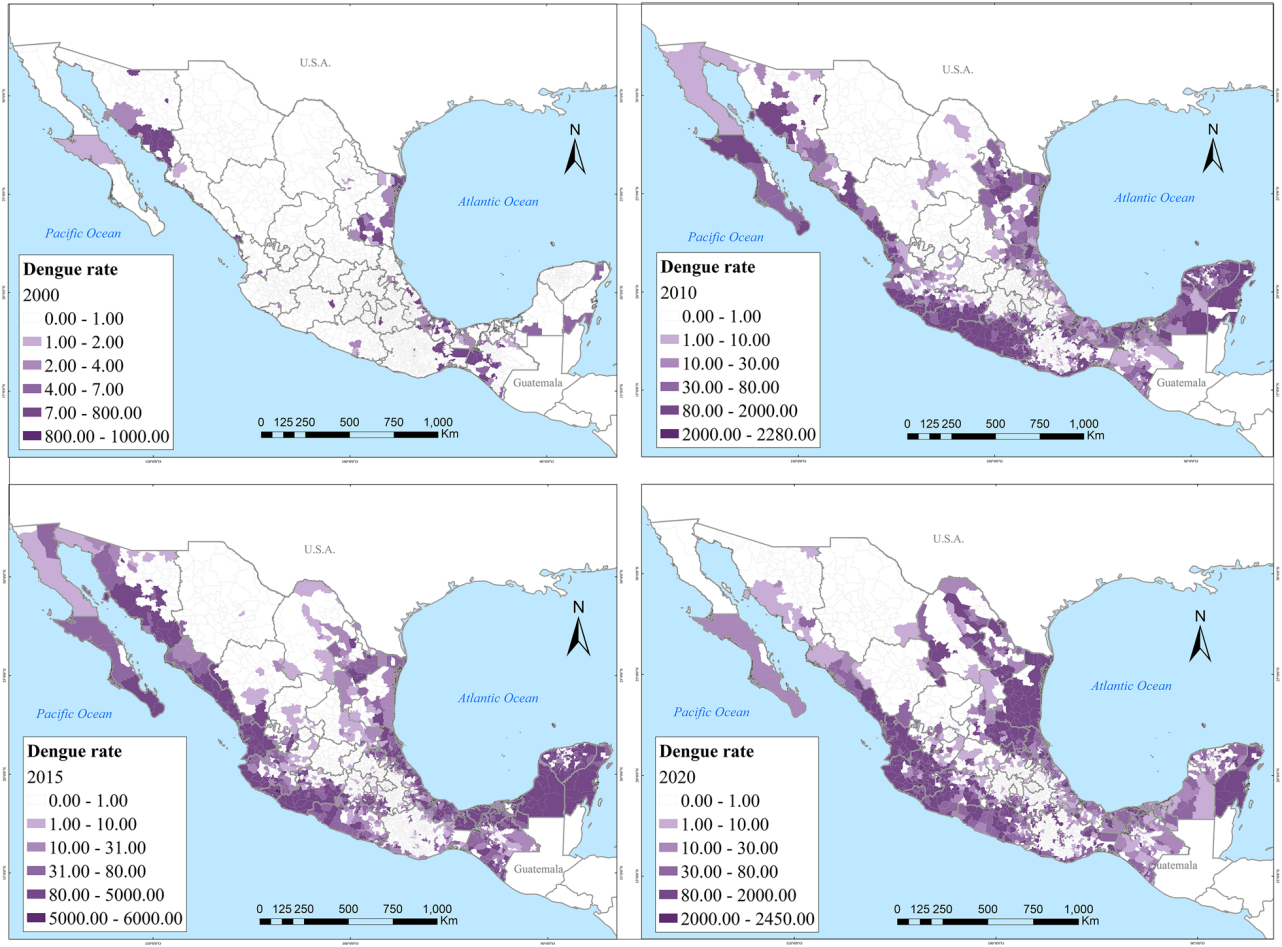
Distribution of dengue in Mexico in 2000, 2010, 2015 and 2020

**Fig. 4:**
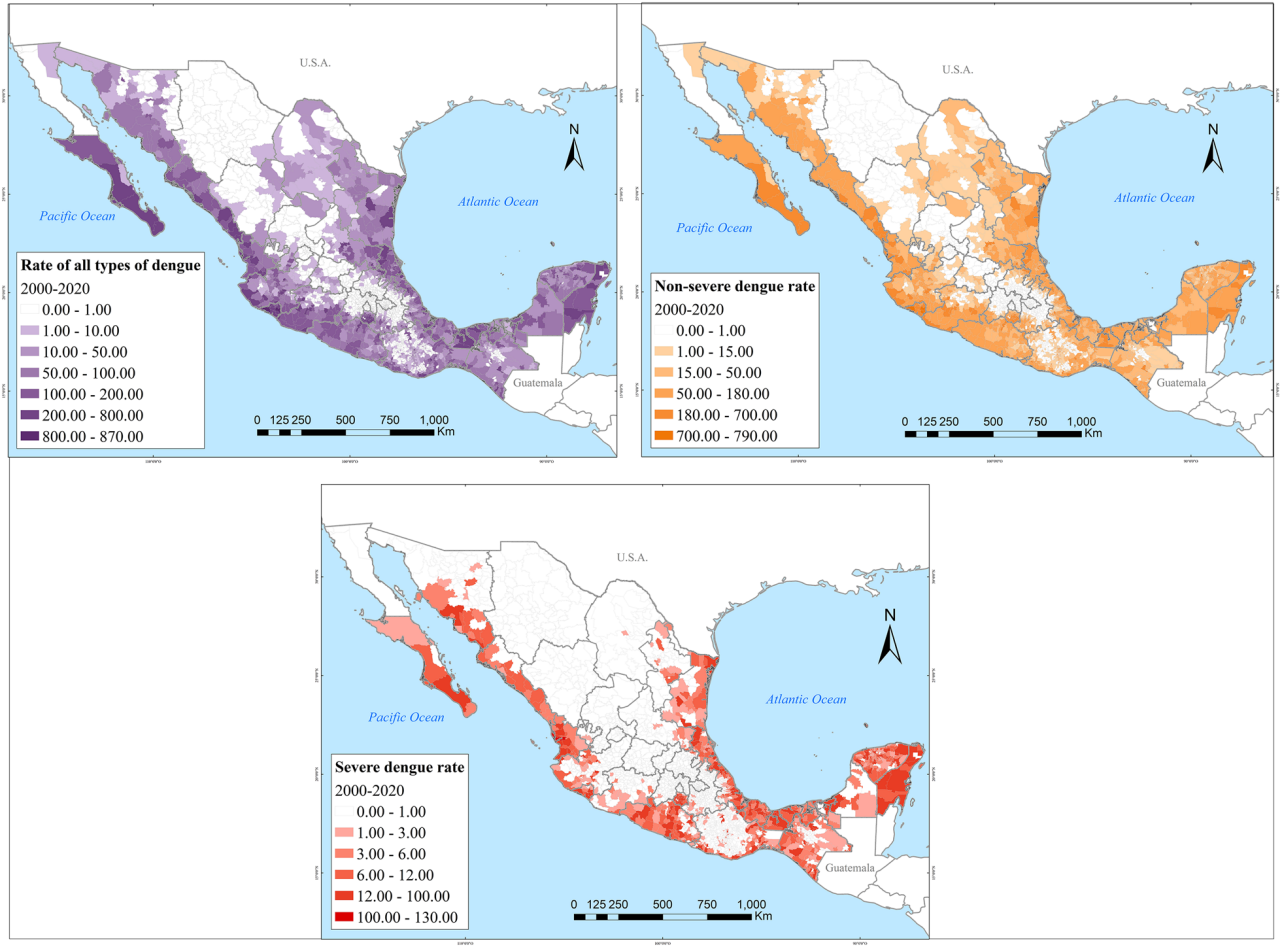
Distribution of severe and non-severe dengue in Mexico during 2000–2020

**Fig. 5:**
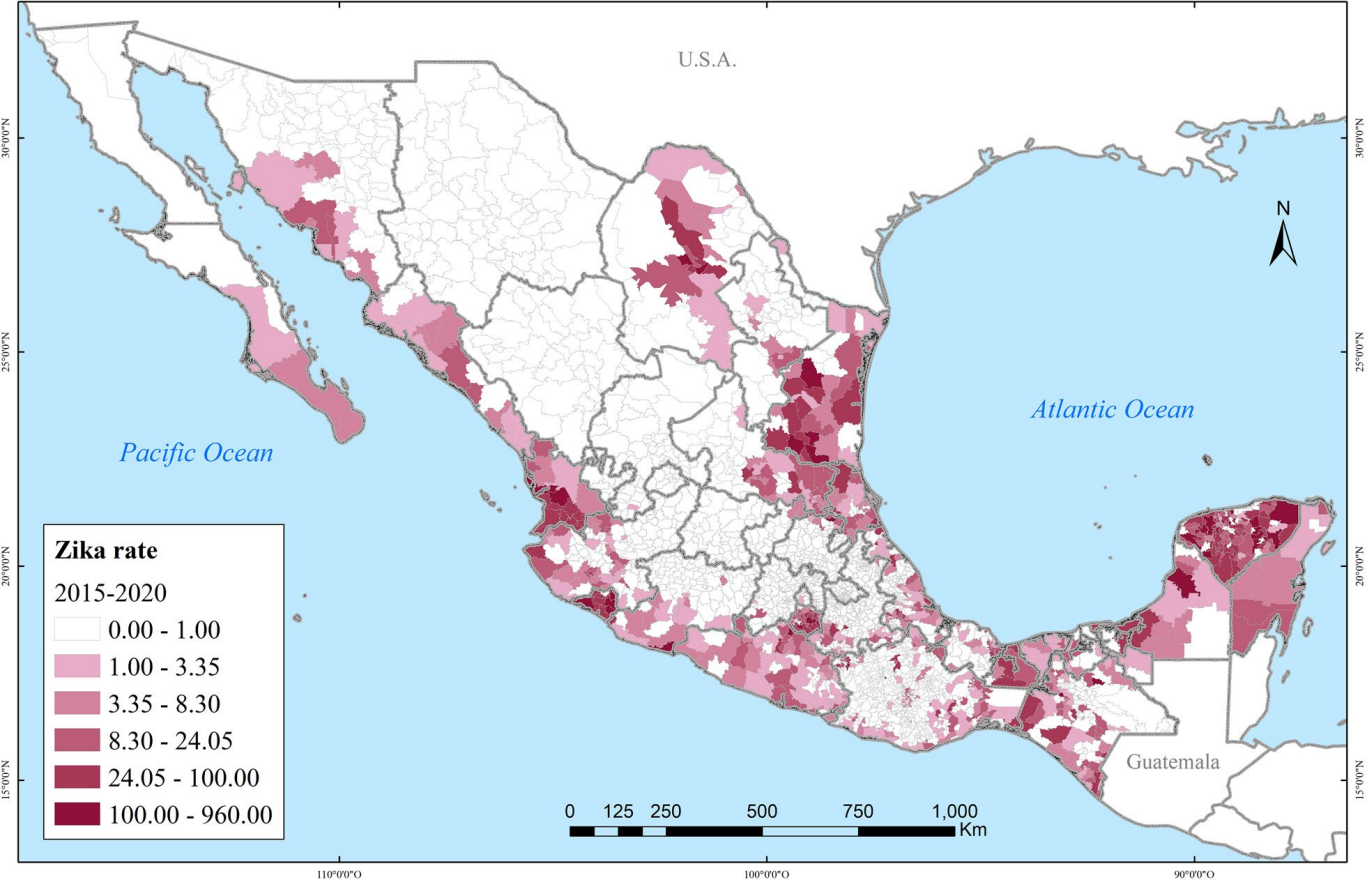
Distribution of Zika in Mexico during 2015–2020

**Fig. 6:**
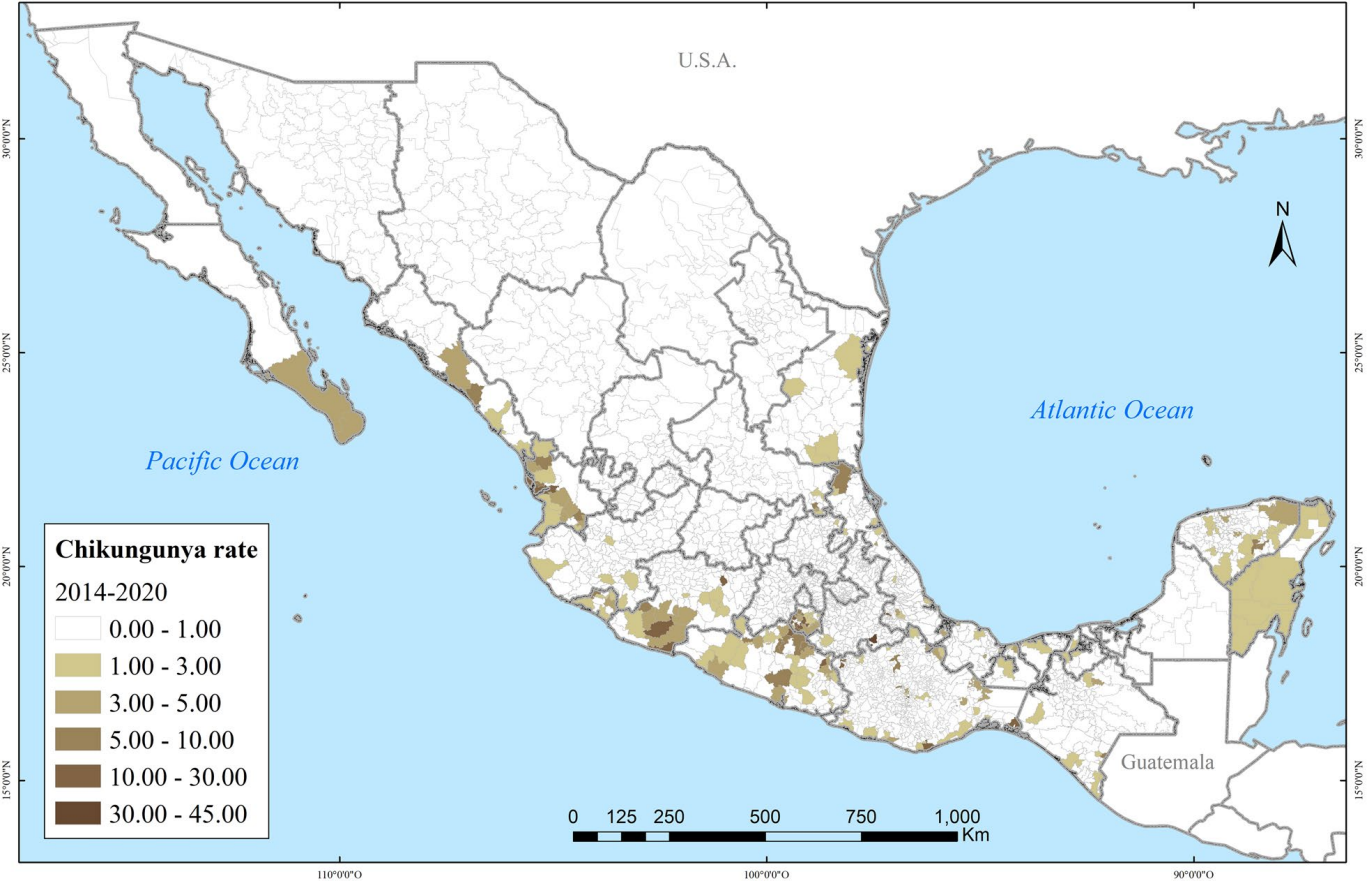
Distribution of chikungunya in Mexico during 2014–2020

Spatially, dengue also rapidly spread in the country. In 2000, dengue affected about a dozen Mexican states. By 2020, at least one case of dengue had been reported across all 32 Mexican states, albeit the highest incidence concentrated on the Yucatán Peninsula, the Gulf, and the Pacific Coasts (Fig. 3). The closer to the coastlines, the incidence of severe dengue intensified (Fig. 4). Zika followed a very similar spatial distribution to that of dengue, clustered in coastal areas (Fig. 5). Chikungunya similarly was found along the coasts, but more sparsely clustered and particularly along the Pacific and the Yucatán Peninsula (Fig. 6).

## Method and data

### Climate-driven mosquito-borne viral suitability index P

To identify the areas and seasons most at risk of mosquito-borne disease transmission, Obolski et al. [18] derive a suitability index, called index P, by modelling the transmission potential of a pathogen. The mathematical derivation of such transmission potential is summarised in two key components: the basic (*R0*) and effective (*Re*) reproduction numbers. *R0* represents the sum of the reproductive potential transmission of each adult female mosquito, *P*_(*u,t*)_, over the number of female mosquitoes per human, *M*, among the susceptible host population (Eq. 1). *Re* also represents reproductive potential but considers the presence of immune hosts hampering transmission, with *S*_h_, *S*_*v*_ standing for the proportion of susceptible humans and mosquitoes (Eq. 2). The overall potential mosquito-borne transmission is summarised in index *P*, as shown in Eq. (3).

1 $${R0}_{\left(u,t\right)}=\sum_{n=1}^{M}\frac{{a}_{(u)}^{v 2}{\phi }_{(t)}^{v\to h}{\phi }^{h\to v}{\gamma }_{(t)}^{v}{\gamma }^{h}}{{\mu }_{\left(u,t\right)}^{v}({\sigma }^{h}+{\mu }^{h})({\gamma }^{h}+{\mu }^{h})({\gamma }_{(t)}^{v}+{\mu }_{(u,t)}^{v})}={MP}_{(u,t)}$$

2 $${Re}_{\left(u,t\right)}={R}_{0\left(u,t\right)}{S}_{h}{S}_{v}$$

3 $${P}_{(u,t)}=\frac{{a}_{(u)}^{v 2}{\phi }_{(t)}^{v\to h}{\phi }^{h\to v}{\gamma }_{(t)}^{v}{\gamma }^{h}}{{\mu }_{\left(u,t\right)}^{v}({\sigma }^{h}+{\mu }^{h})({\gamma }^{h}+{\mu }^{h})({\gamma }_{(t)}^{v}+{\mu }_{(u,t)}^{v})}$$

The estimation of *R0* depends on defining priors for all the eight parameters. Four of these parameters are climate independent. These are the human life span 1/*μ*^*h*^; the transmission probability from an infected human to mosquito per bite ${\phi }^{h\to v}$; the human infectious period 1/*σ*^*h*^; and the human incubation period 1/*γ*^*h*^. The other four parameters are climate dependent: the life span of adult mosquitoes 1/${\mu }_{(u,t)}^{v}$, the extrinsic incubation period 1/${\gamma }_{t}^{v}$, the daily biting rate ${a}_{(u)}^{v}$ and the probability of transmission from an infected mosquito to human per bite ${\phi }_{(t)}^{v\to h}$. All these climate-dependent parameters are dependent on humidity (*u*) and temperature (*t*) and have been previously determined in experimental laboratory studies [18]. The climate-dependent parameters can also be extended to include the role of precipitation, as done in this paper. In this case, precipitation replaces humidity in the effects over egg hatching success.

*R0, Re* of a mosquito-borne virus have seasonal oscillations dependent on changes in climate conditions and other parameters such as the number of adult female mosquitoes per human (*M*). Obolski et al. [18] explain it is rare to have accurate estimations of *M* for regions or mosquito species of interest. However, theoretically, the potential for outbreaks is determined by the epidemic thresholds of *R0* > 1 or *Re* > 1. Thus, if at least one female mosquito exists per human (*M* >  = 1) and *P*_(*u,t*)_ > 1, then *R0* = *MP*_(*u,t*)_ > 1 and epidemic growth is possible.

The index P, shown in Eq. (3), is derived by fitting a dynamic model within a Bayesian Markov chain Monte Carlo framework, assuming only one main human host. In this Bayesian model, the index P is estimated by defining eight priors in the expression of *P*_(*u,t*)_ which establish the relationship between meteorological variables, mosquitoes, and host parameters such as viral incubation periods, adult mosquito lifespan, and mosquito bite rate. All these priors have been estimated in the literature. Table 3 in the Appendix lists the systematic studies that Obolski et al. [18] sourced to determine these parameters along the distribution of parameters used as priors. Here, we use the same eight priors that Obolski et al. [18] used to estimate the index P for Brazil.The four climate dependent parameters used in Eq. (3) have been determined in experimental laboratory studies and estimates of entomological data under various climate conditions [24–30]. The other climate independent parameters such as human incubation period and human infections that Obolski et al. [18] used were established based on systematic reviews of Latin American studies [27, 31, 32]. We did not change the parameter of human-life expectancy as this parameter, set for Brazil in the MVSE R package, is very similar to the one in Mexico. However, we did perform sensitivity analysis, and like other recent studies, concluded that the index P is robust to a range of priors [15]. In the Results section we discuss this sensitivity analysis.

The index P is a summary statistic that measures the transmission potential of an adult female mosquito. To put it simply, the index P is the likely average number of new infections generated by a single infected adult female in a susceptible host population. Thus, a key advantage, and difference from other suitability indices not simply based on vectorial capacity [21, 33], is that the numerical scale of the index P has a direct biological interpretation. Moreover, in absolute value, the index P is informative for the timing and amplitude of potential transmission when assessed locally in time or between regions [18].

### Estimating the suitability index P for Mexico

Another advantage of the index P is that it can be estimated using readily available climate data and the freely available Mosquito-borne Viral Suitability Estimator (MVSE) R-package. We use the latest release of the MVSE R-package, version v1.01r. Detailed technical features of this R-package can be found in Obolski et al. [18]. These authors estimated their index P for Brazil using an earlier MVSE R-package version (v0.33). In this paper, we use the most recent version of their R-package, version v1.01r, which, unlike previous versions, adds precipitation as one of the key components to estimate the index P. This package also allows the user to set the values of the priors depending of the host–pathogen system analysed.^1^ We used the default priors that Obolski et al. [18] used, but as discussed in the Results section our results remain robust to using other priors.

### Data

To estimate the index P for Mexico, we use daily data of temperature, relative humidity, and precipitation obtained from the 188 automatic meteorological stations of the National Meteorological Service of Mexico for 2000–2020. These stations take measurements of the meteorological variables automatically using electrical and mechanical devices that are later sent via satellite and collected by the personnel of the Meteorological Service. The stations are distributed across the country and the data they collect do not correspond to municipality administrative boundaries. However, the data available from these automatic statics allowed us to interpolate climatic data for all the 2469 municipalities in the country.

Automatic meteorological stations are less densely distributed than alternative non-automatic meteorological stations and observatories. Nonetheless, automatic stations still offer excellent coverage of the country’s weather conditions at a small-area level. Moreover, the automatic stations are the only ones that measure the relative humidity on the surface, which is one of the key data required to estimate the index P. The relative humidity is captured even in different time intervals daily, which is ideal for the daily estimate of the index P in this study. Automatic stations also offer much wider data availability suitable for daily analysis than other databases that provide perhaps a broader spatial resolution but with limited-time series. WorldClim for instance does not have direct data on humidity, it only offers saturation vapour pressure information which could be used to infer the levels of relative humidity. Nonetheless this potential inference and wider spatial resolution could increase the measurement error and potential degree of uncertainty to the index P daily estimates.

In the next section, we illustrate the dynamics of the index P for three relevant cities daily during the period 2000–2020. This daily analysis allows us to assess the index’s sensitivity to seasonal changes in climate conditions. Then, we provide a much broader picture of the distribution of the index P across all the Mexican territory by focusing on seasonal changes of the index P over time. In Additional file 1: Table S1, we present the monthly index P for each automatic meteorological station, aggregated at state level, in the country during 2000–2020. Our analysis is performed over a regular grid of Mexico, in which each pixel represents an area of approximately 50km^2^. This mesh results from the Inverse Distance Weighted (IDW) interpolation as calculated by the QGis package commonly used to interpolate climatic data. IDW determines cell values using a linear weighted combination of sample points, where the weight is a function of the inverse distance raised to a mathematical power. Points closer to each other are given more weights as they are assumed to be more similar and more influential than those at a greater distance [34]. As power we set the default parameter in the QGis package, which is equal to two. The magnitude of this parameter is in practice not very important for our results because the robustness of interpolation depends more on the density of the data and the resolution of the geographic area analysed, in our case for the whole country. There are other methods, such as Kriging, but these are more suitable to interpolate other types of data.

## Results

### Index P dynamics in three case studies

Before we present the trends of the index P and mosquito-borne diseases for the entire country, we pause to analyse in more detail three cities: Acapulco, Cancún and Mexico City. We focus on these three cities because they have important differences in climatic conditions, geographical location, arbovirus incidence and are subject to distinct and very high degrees of human migration patterns. These differences make these cities ideal for assessing the robustness of the index P in estimating transmission risks on a daily basis during 2000–2020.^2^

Acapulco, on the east Pacific coast, has a tropical wet and dry climate characterised by high temperatures with minimal variation and variable levels of precipitation [35]. Cancún has a tropical savanna climate on the Yucatán Peninsula [36]. Both these cities are among the most important domestic and international tourism centres in the country, and have high dengue incidence, albeit with peaks in different seasons. Mexico City, the capital and most populated city in the country, has a subhumid mild climate. This city is a particularly interesting case to analyse because its high elevation, 2240 m above sea level, is above the elevation ceiling that typically allows the *Aedes* mosquitoes to proliferate [37]. Nonetheless, climate warming could over time place high-elevation cities at increased risk of dengue transmission; and in the Americas the *Aedes* have been found in other similar high-altitude areas of 2200 m [38].

**Fig. 7:**
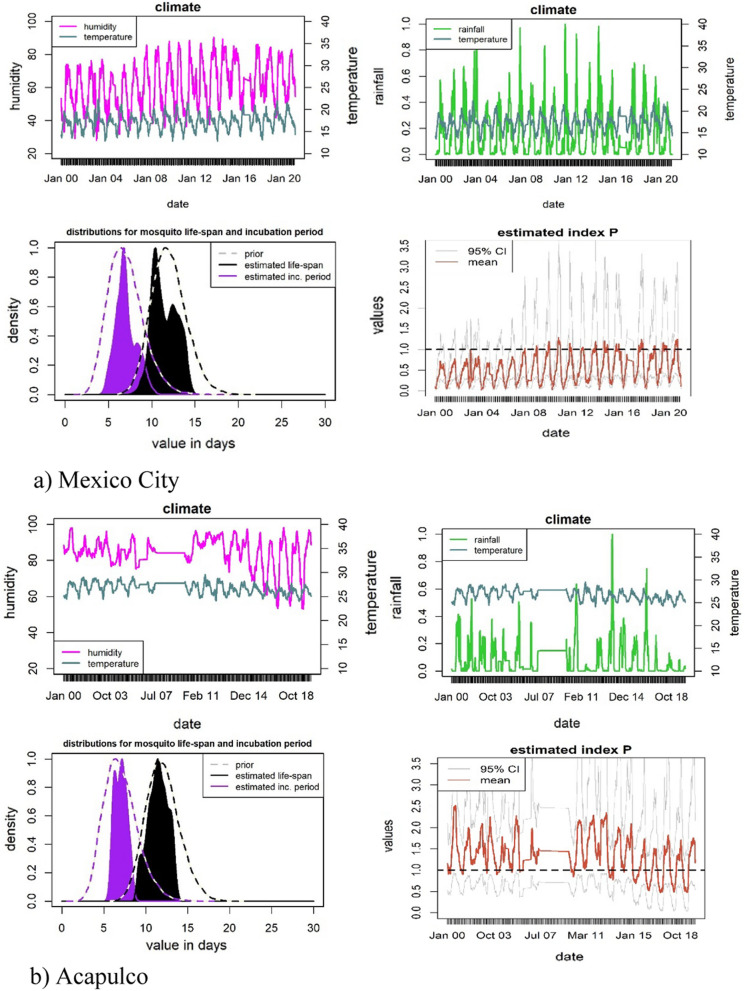

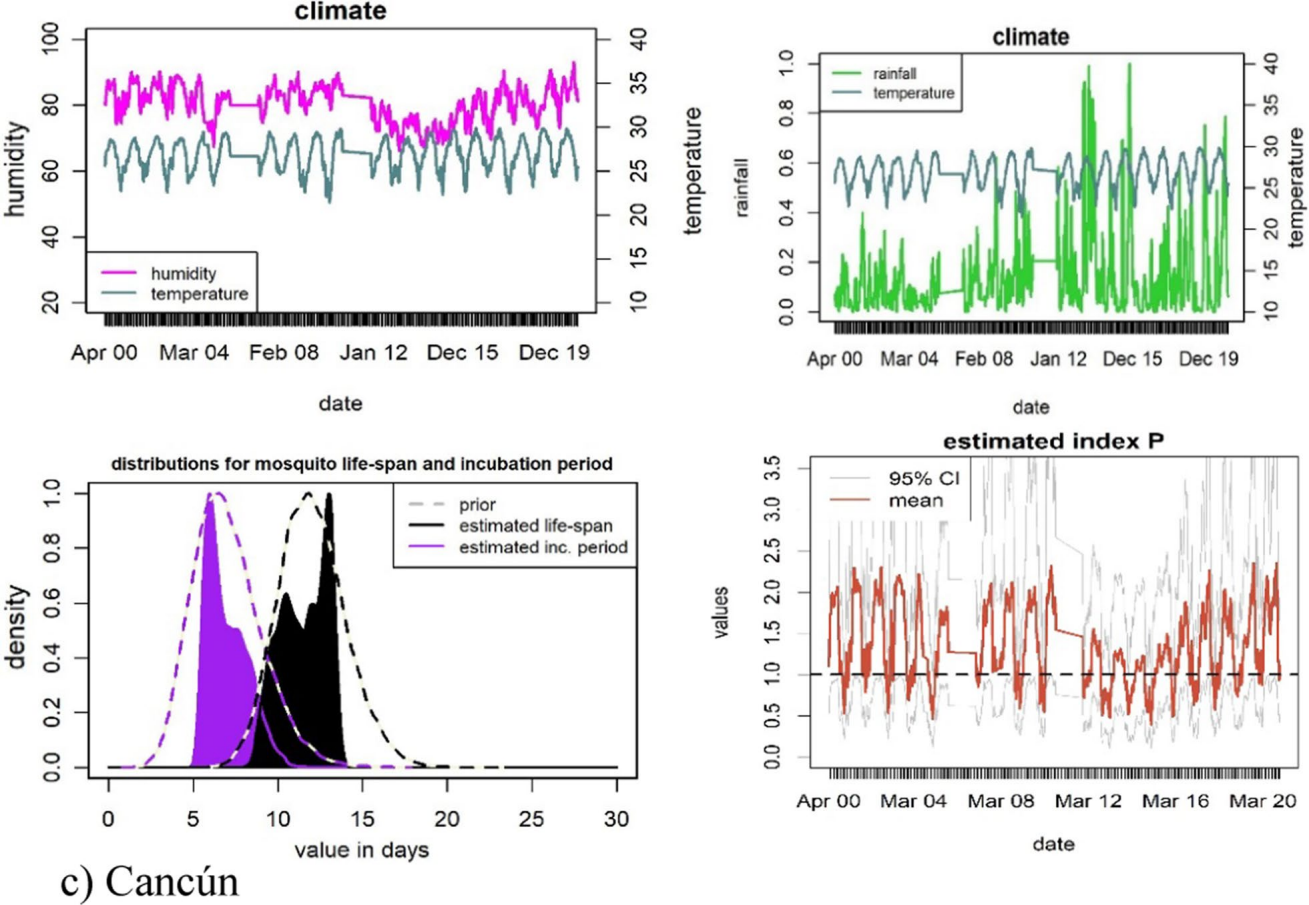
Climate, entomological priors, index P and 95% confidence intervals for Mexico City, Acapulco, and Cancún 2000–2020

For each of our three case studies, Fig. 7 displays the daily climate patterns (temperature, humidity, and precipitation), the distribution of the entomological priors and the estimated index P.^3^ Following the literature, we assume the entomological priors of the mosquito lifespan and incubation period to be the same across all the three cases [18]. As expected, Acapulco and Cancún have consistently higher indices P than Mexico City’s (with averages of 1.35, 1.32 and 0.54, respectively). These indices P predict that if there was one female mosquito per human, Acapulco and Cancún would be more susceptible to outbreaks of mosquito-borne diseases than Mexico City, where the index is less than one for most of the year. Acapulco and Cancún present higher levels of humidity and temperature and are subject to more irregular trends in all climate conditions than Mexico City. These patterns suggest that the daily index P for Acapulco and Cancún have more irregularities in their seasonal patterns. In contrast, Mexico City displays a more stable seasonal pattern during 2000–2020. These risks of transmission are well in line with the epidemiological profiles of each city. Acapulco, Cancún and Mexico City have a markedly different incidence of dengue per 100,000 inhabitants, with an average of 12.06, 8.98, and 0.015 respectively during 2000–2020. In fact, Acapulco has one of the most severe and persistent dengue-incidence profiles in the country, whilst Cancún has in recent years increased from medium to high levels of dengue [39]. Mexico City’s low index P also corresponds with its low incidence of dengue, chikungunya and Zika, typical of local climate conditions of high-altitude areas, which are not conducive to the endemic presence of *Aedes* mosquitoes.

**Fig. 8:**
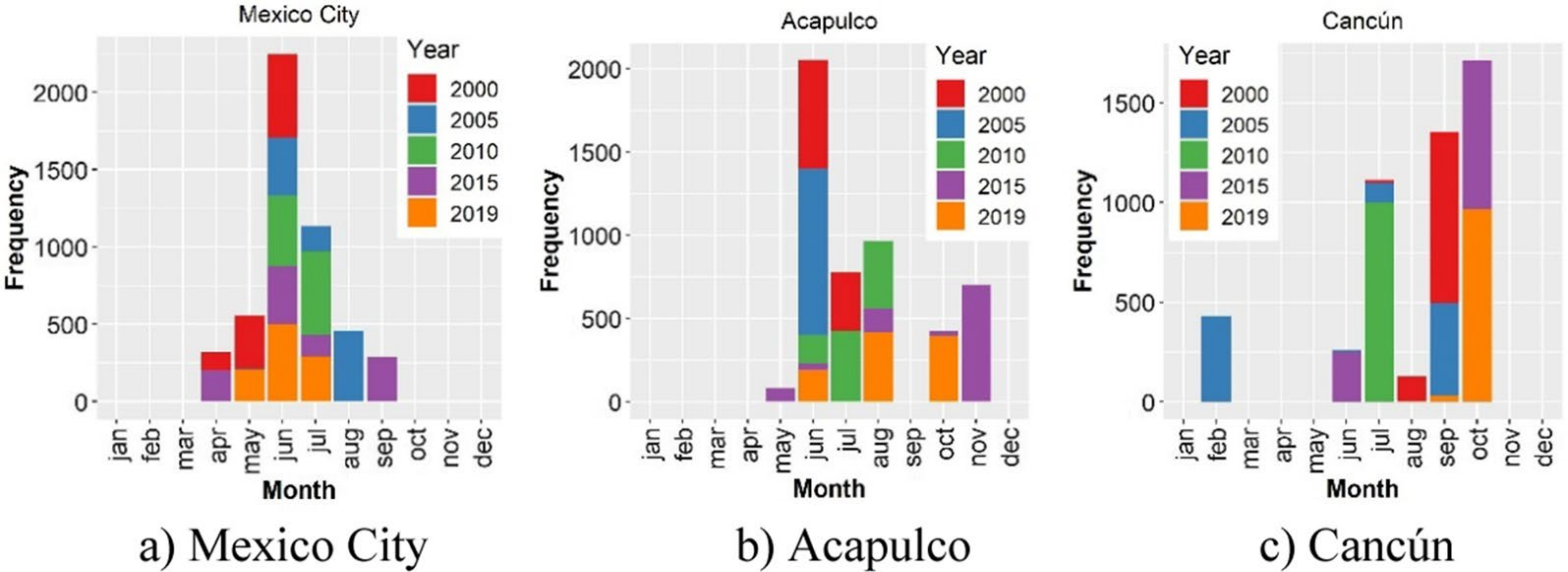
Peak distribution of the index P

The timing of when the index P reaches its maximum can be used to determine the timing of the highest mosquito-borne disease transmission potential. For visual simplification, Fig. 8 shows the month for which the index P reached its peak across all the simulations for the years, 2000, 2005, 2010, 2015 and 2019. Over this sub-period analysed, the index P in Mexico City tended to peak in June, reflecting very marked seasonal behaviour. For Acapulco and Cancún, their indices P tended to reach their maximums in June and October, respectively. Nonetheless, the timing of when the index P peaked displayed a high degree of variance.

**Fig. 9:**
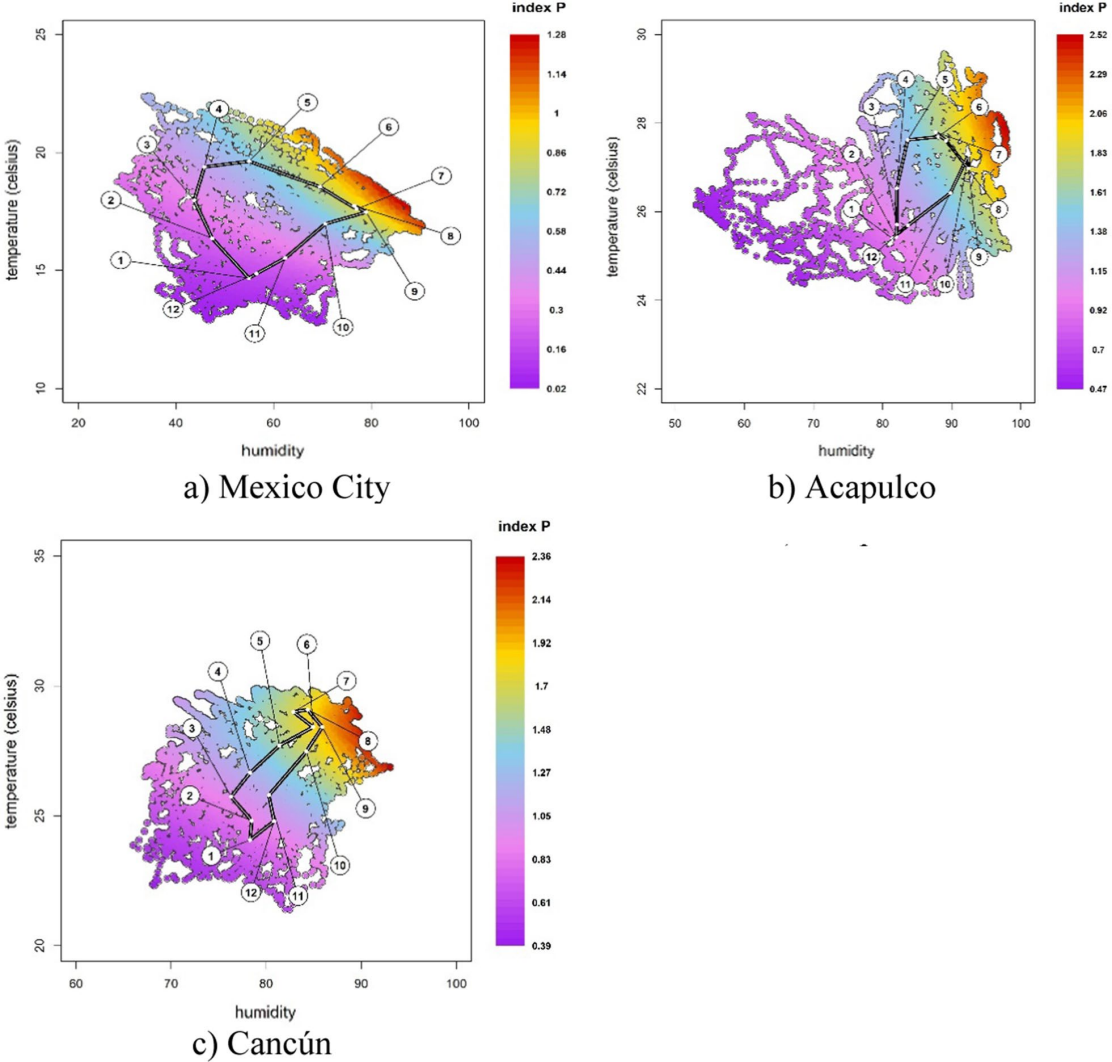
Each point represents a humidity-temperature combination recorded in the climate data used as input for the index estimation. Temperature is measured in degrees Celsius. The white dots over the black link mark the mean for each month, while the floating circles indicate which month it refers to

Another key feature of the MVSE R-package is that it offers a visual representation of the sensitivity of the estimated index P to changes in climate conditions. Figure 9 depicts the humidity and temperature on the x- and y-axis, respectively, for all the combinations in the climate data. The different colours in Fig. 9 represent different values of the index P. The dots that form a ring shape within the figure represent the average values of the climate data for each month. The floating circles with numbers ranging from one to twelve indicate what month the average values refer to. Figure 9 shows that suitability in Mexico City follows a clear and gradual trend along the months in a year, while Cancún presents abrupt changes reflecting lower stationarity. The lower stationarity hinders the identification of the month of highest transmission risk. Acapulco behaves somewhere in between the other two case studies.

As mentioned earlier, the estimation of the index P relies on defining priors for eight parameters. To assess how robust the index P is to these priors, we performed sensitivity analysis. We changed the value of some of our initial priors, taken from Obolski et al. [18] for the prior of human incubation period (from mean = 5.8, sd = 1, to mean = 5.0, sd = 1), or the mosquito life expectancy (from mean = 12, sd = 2, to mean = 14, sd = 3), or both these priors simultaneously. We changed the value of these priors as there is also support in the literature for these parameters which have been used recently in the estimation of the index P for the Dominican Republic [15]. In our sensitivity analysis we found no statistically significant differences with respect to the set of priors we used originally, as shown in Fig. 13 in the Appendix. This figure shows the value of index P using our original priors and the value of the index P when changing the human incubation period (left-hand side panel), or the mosquito life expectancy (middle panel), or both these parameters simultaneously (right-hand side panel). Figa. 13 also shows the resulting 95% confidence intervals for the estimated indices P. In most cases, the only noticeable change, is a slight increase in the upper confidence interval, particularly when we change the mosquito life expectancy parameter.

### Index P spatiotemporal characterisation across Mexico

In this section, we provide a broader picture of the distribution of the index P across all the Mexican territory. As mentioned earlier, automatic meteorological stations offer the key advantage of measuring on the ground local climate conditions daily. Our choice of using these automatic measurements comes at a cost. Unfortunately, not all regions in the country had an automatic meteorological station nearby during 2000–2010.^4^ Nonetheless, the number of automatic stations sharply increased after 2010, achieving good national coverage during 2010–2020. For this reason, in this section and the next one, we restrict our analysis to 2010–2020. In Additional file 1: Table S1, we present the index P for all available automatic meteorological stations, aggregated at state level, during 2000–2020.

**Fig. 10:**
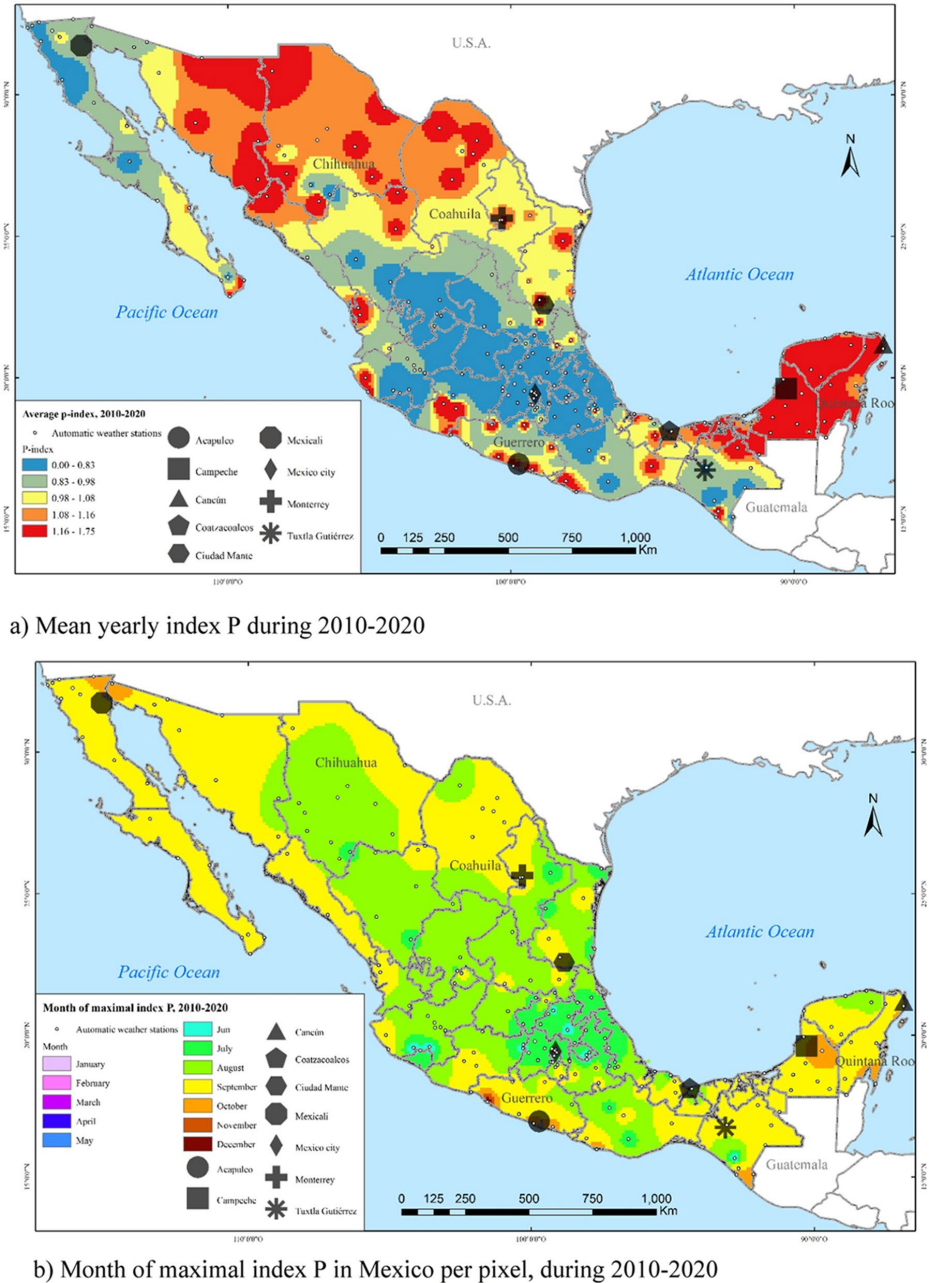
Spatial–temporal characterisation of the index P in Mexico per pixel, during 2010–2020

Figure 10a, shows the average index P across all the Mexican territory during 2010–2020. This figure depicts the values of the index P by quintiles. As mentioned earlier, the risk of transmission increases when the index P takes a value greater than one, and the risk decreases when the index P takes the value of less than one. The regions with the highest index P (index > 1.16, shown in red) are in the southeast (Tabasco and the Yucatán peninsula), the Pacific coast, and in some northern states (Sonora, Chihuahua and Coahuila). The intermediate values of the index P (index between 0.98 and 1.16, shown in yellow and orange) are located in the Tehuantepec Isthmus and in some northern states. The index P with its lowest value (index < 0.98, blue and green), hence with the lowest transmission potential, is found in the centre of the country and the peninsula of Baja California.

The month where the index P reaches its maximum value is shown in Fig. 10b. It stands out that the maximum peak per month does not always occur in the same regions, as it is mainly influenced by temperature variability. The index P peaks in July in the centre and centre east. For most of the rest of the country, the index P reaches its maximum value in August or September, where the transition from summer to autumn begins.

**Fig. 11:**
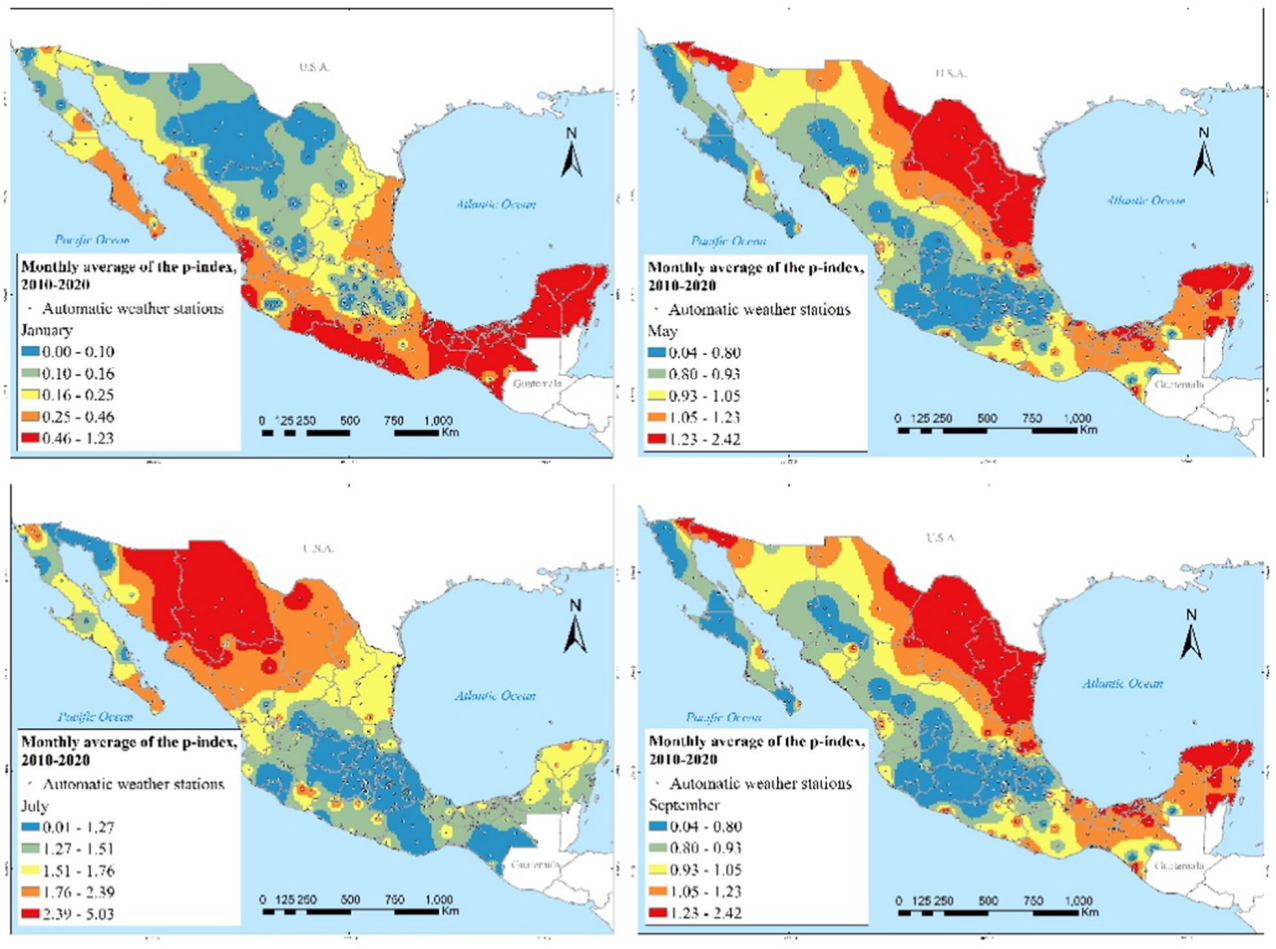
Mean of index P during 2010–2020 in January, May, July and September

Figure 11 shows the monthly average of the index P during 2010–2020 for selected months. During January, the southeast, Tehuantepec Isthmus and the coasts of Michoacán and Guerrero stand out with the highest transmission potential. In May, the highest transmission potential occurs in the northeast region. In July, the highest transmission risk shifts to the northwest of the country, standing out the states of Sonora and Chihuahua. In September, the highest transmission risk is for northeast states, particularly Coahuila, Nuevo León, and Tamaulipas, as well as Yucatán in the southeast.

### Correlation between the index P and mosquito-borne diseases in Mexico

In addition to practicality, good suitability indices must also be correlated to the phenomenon they intend to measure. Thus, next we assess the degree of correlation between dengue, chikungunya and Zika incidence and the index P for all the 2469 municipalities in Mexico and for nine selected cities during 2010–2020. These nine cities have been selected to provide a granular view across the territory, given their varying levels of arboviral infections and differences in socio-economic characteristics. These cities are in the north of the country (Ciudad Mante, Mexicali, and Monterrey), centre (Mexico City), southeast (Campeche and Tuxtla Gutiérrez), and various coasts (Acapulco, Cancún, and Coatzacoalcos). The geographical location of these nine cities is shown in Fig. 10. For each of these nine cities we estimate the Pearson correlation between the index P and dengue, Zika and chikungunya during 2010–2020. Like Obolski et al. [18], for each city, we estimate its Pearson correlation coefficient between the average index P for each month during 2010–2020 and its monthly average incidence of each arboviral disease (measured in natural logarithm) during 2010–2020.

**Fig. 12:**
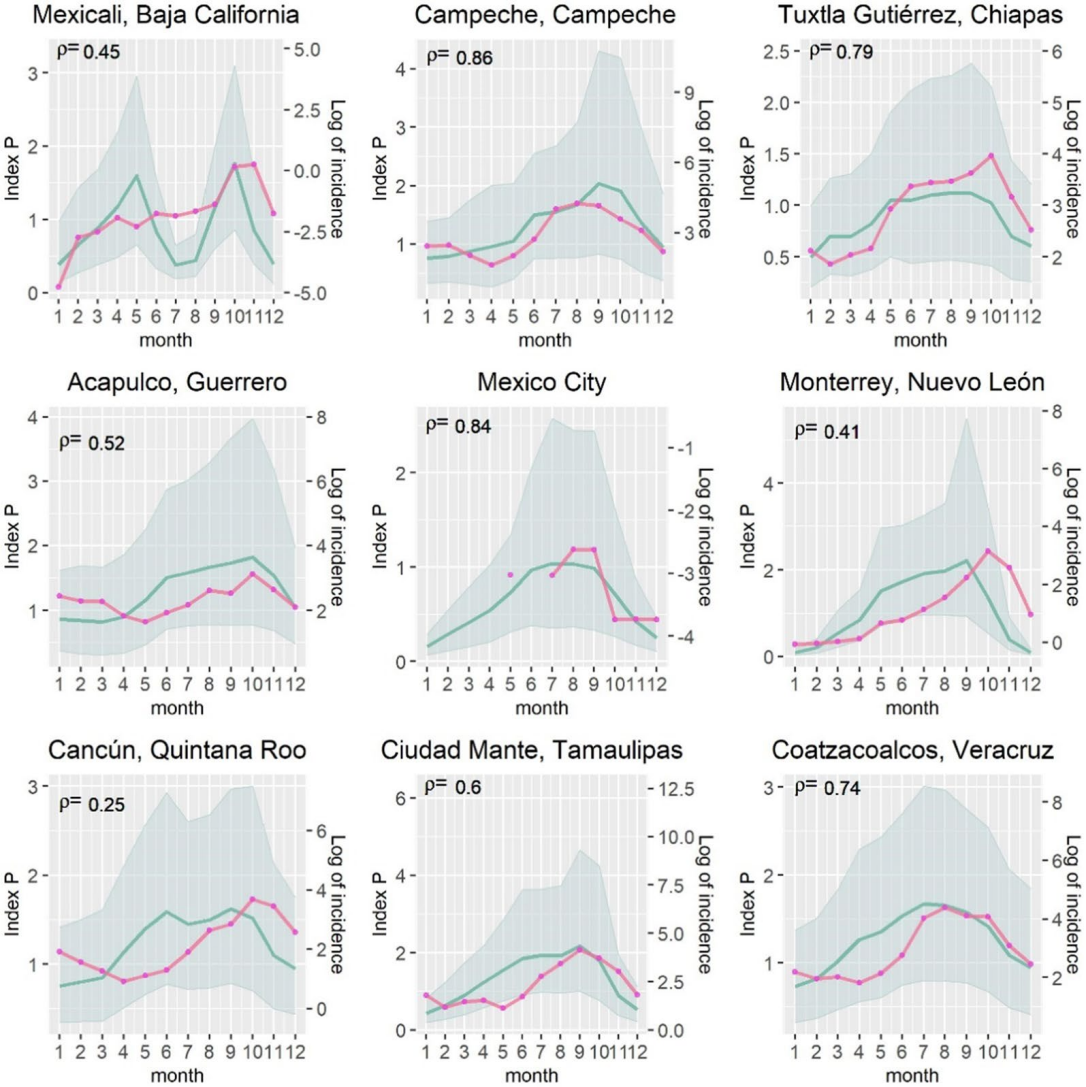
Correlation of index P and dengue incidence for selected nine cities. Monthly averages of the index P are shown in blue and average incidence is shown in the dotted pink line. Shaded areas correspond to the 95% confidence intervals of the index. Pearson’s correlation is shown in each subplot

**Fig. 13:**
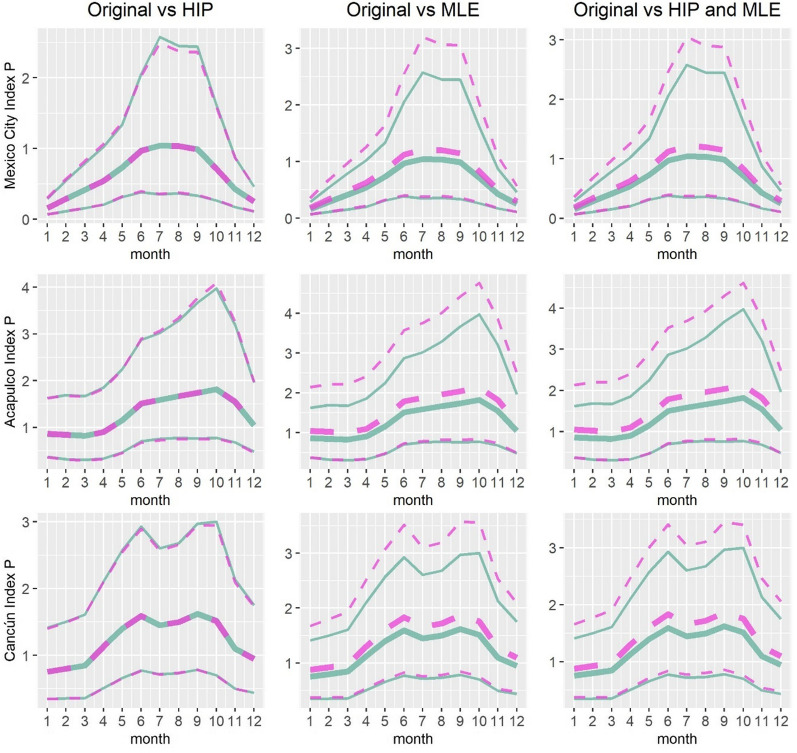
The blue solid line shows the average monthly index P and its 95% confidence interval (upper and lower thresholds) during 2010–2020 when using the same priors as Obolski et al. [18]. The pink dashed line shows these estimates changing two parameters to the values used by Petrone et al. [15]. The left-hand side panel shows the estimation when changing the Human Incubation Period (HIP) parameter. The middle panel shows the estimations when changing the Mosquito Life Expectancy (MLE) parameter. The right-hand side parameter shows the estimation when changing both these parameters simultaneously

Figure 12 depicts for each of the nine cities its monthly average index P, its monthly incidence of dengue (measured in logarithm) during 2010–2020, as well as the Pearson correlation between these variables. This correlation index is positive and ranges between 0.25 (Cancún) and 0.86 (Campeche). The correlation is much higher for southern and coastal cities that tend to have a high incidence of dengue, with the exception of Cancún perhaps due its very high flow of international and domestic tourism. The correlation is lower for northern cities that typically have low levels of dengue (Mexicali and Monterrey). These results suggest there might be other relevant factors, such as population mobility, density and socio-economic characteristics that explain the low incidence of dengue in some cities, which the index P does not consider. Nonetheless, overall the correlation between the index P and dengue are similar to the results obtained by Obolski et al. [18] for several cities in Brazil.

**Fig. 14:**
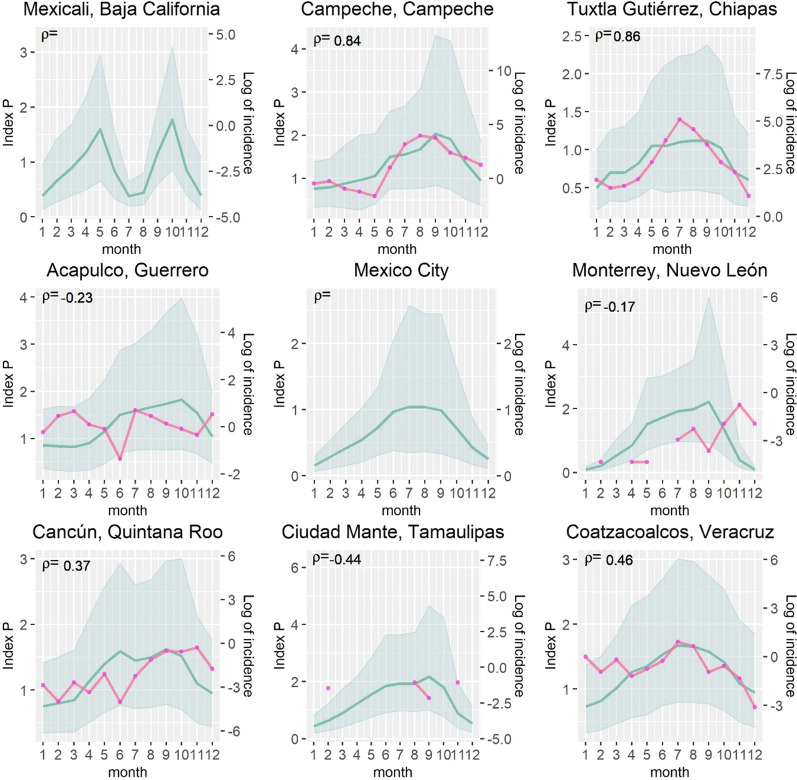
Correlation between index P and chikungunya. Monthly averages of index P are shown in blue, and the average incidence of chikungunya is shown in pink. Shaded areas correspond to the 95% confidence intervals for the index. For each city, the index averages are computed for any year between 2010 and 2020 for which climate data is available. The geographical localisation of these cities is shown in Fig. 10. Pearson’s correlation is shown in each subplot

**Fig. 15:**
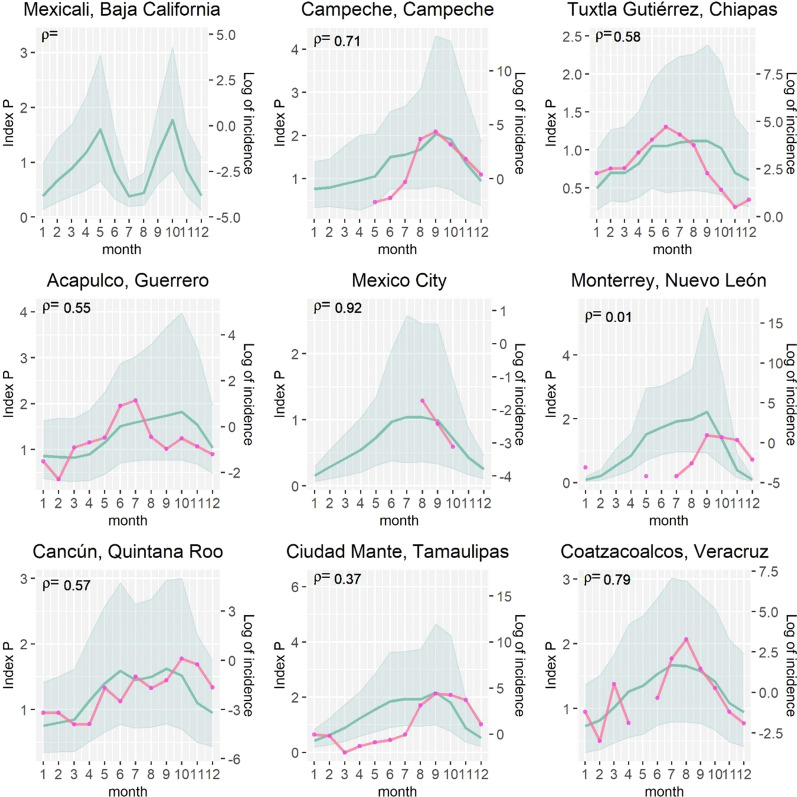
Correlation between index P and Zika. Monthly averages of index P are shown in blue, and the average incidence of Zika is shown in pink. Shaded areas correspond to the 95% confidence intervals for the index. For each city, the index averages are computed for any year between 2010 and 2020 for which climate data is available. The geographical localisation of these cities is shown in Fig. 10. Pearson’s correlation is shown in each subplot

To analyse further the transmission risk potential predicted by the index P, Figs. 14 and 15, in the Appendix, show the correlation between the index P and chikungunya and Zika for the nine selected cities since they appeared in the country in 2014 and 2015 respectively. The incidence of both diseases has rapidly declined and is more clustered in specific regions. However, in the cities where there is chikungunya or Zika or both, and where it is possible to estimate the correlation with the index P, the correlation is relatively strong. For chiungunya the correlation ranges from -0.44 (Ciudad Mante) to 0.86 (Tuxtla Gutiérrez). For Zika the correlation with the index P ranges from 0.01 (Monterrey) to 0.92 (Mexico City).

There is substantial variability in the presence of Zika and chikungunya across the nine cities as these diseases are not widespread but concentrated in certain parts of the country only. This partly explains why the correlation coefficient between the index P and the rate of Zika and chikungunya shows wider range than the correlation between the index P and dengue. Moreover, the sporadic and scant incidence of Zika and chikungunya provides us with insufficient power to detect a statistically significant correlation between the index P and these arboviruses for each of these nine case studies.

To provide a more global view, Table 1 presents the Pearson correlation between the index P, dengue, Zika and chikungunya for two different scenarios: one considering all the nine cities together, and another considering all the 2469 municipalities in the Mexican territory during 2010–2020. We find that the correlation between the index P and dengue is 0.46 when considering together the data from the nine case studies. This correlation is statistically significant, and higher than when estimating the correlation for all the 2469 municipalities in the country (0.29) as the selected nine case studies tend to have high incidence of dengue (with the exception of Mexico City).

**Table 1:** Correlation between Index P, dengue, Zika and chikungunya incidence for case study and all municipalities in the country 2010–2020

		Dengue	Zika	Chikungunya
Nine selected cities in Mexico	Correlation coefficient	0.46	0.20	0.01
	P-value	0.00	0.34	0.96
	Number of observations	103	78	73
All 2,469 municipalities in the country	Correlation coefficient	0.29	0.23	0.25
	P-value	0.00	0.00	0.00
	Number of observations	12,411	3542	3381

Table 1 also shows that the correlation between the index P and dengue for all the 2469 municipalities in the country (0.29) is of similar magnitude to that of Zika (0.23) and chikungunya (0.25). All these correlations between the index P, dengue, Zika and chikungunya are statistically significant, and sufficiently powered with large number of observations, when considering the data across all the municipalities in the country.

The nine selected cities in Mexico are: Acapulco, Campeche, Cancún, Ciudad Mante, Coatzacoalcos, Mexicali, Mexico City, Monterrey, and Tuxtla Gutiérrez. The number of observations is the number of areas considered multiplied by twelve (as the correlation considers the average rate of mosquito-borne disease for each of the 12 months over the period 2010–2020). Some areas do not report mosquito-borne diseases for some months. For these missing cases the number of observations is reduced, as it is particularly the case for Zika and Chikungunya.

### Hurricane Manuel and index P

Our analysis suggests that the index P offers valuable information on the potential dynamics of mosquito-borne risk transmission during a given year or for a long-time series. Another possible application of the index P could be to assess changes in transmission potential due to sudden weather shocks such as hurricanes. Adult mosquitoes do not generally survive during the high wind speed associated with hurricanes. However, a disease outbreak of dengue, Zika, and chikungunya might follow as hurricanes might cause significant property damage and increase precipitation that makes it more likely for mosquitoes to breed [40–42]. To assess to what extent hurricanes affect the index P, Table 2 shows the changes in monthly average index P, temperature (Celsius), monthly average humidity (a percentage that ranges between 0 and 100), and the monthly average precipitation (in millimetres), associated with Hurricane Manuel that affected Mexico in September 2013. Manuel was the first eastern North Pacific tropical cyclone to make landfall in mainland Mexico, redevelop over water, and then become a hurricane. Manuel brought heavy rains and floods to large parts of the Pacific coast, resulting in 123 deaths and 4.2 billion US dollars in damage, with the biggest impacts in Guerrero [43]. Over 30,000 homes were damaged in that state alone, and 46 rivers overflowed. Table 2 shows the sharp increase in rainfall that Acapulco in Guerrero experienced during September 2013.^5^ The index P, as a result, increased during September 2013. This increase in the index P also coincided with the rise of dengue incidence in Acapulco during that month. This evidence suggests that the index P reasonably predicts how climate changes can lead to changes in potential transmission. It is worth noting that the index P seems to be more sensitive to changes in temperature and humidity. For instance, in September 2012, Acapulco did not experience a hurricane in the previous year. Nonetheless, there were statistically significant higher levels of humidity and temperature reflected in a higher index P and higher dengue incidence than the ones experienced in September 2013 where Hurricane Manuel affected Guerrero.

**Table 2:** Climate conditions, Index P and dengue incidence in Acapulco during July-November 2012 and 2013

Month	Temperature Celsius	% Humidity	Rain Millimetres	Index P	Dengue incidence	Temperature Celsius	% Humidity	Rain Millimetres	Index P	Dengue incidence
	Year 2012					Year 2013				
July	28.64	91.67	0.01	2.07	10.92	27.05	90.19	0.05	1.62	8.99
August	27.91	94.21	0.05	2.18	35.36	27.03	89.64	0.05	1.65	9.85
September	27.90	94.94	0.04	2.23	35.49	25.97	95.98	0.23	1.84	9.36
October	27.91	95.80	0.03	2.24	63.65	26.66	95.06	0.08	1.95	26.84
November	26.38	89.71	0.01	1.52	37.72	26.43	95.36	0.00	1.91	8.62

Our evidence suggests that the index P is a good tool to assess increased risk of transmission which could alert policymakers which months, seasons, and areas could be at increased risk of mosquito-borne diseases due to changes in climatic factors. However, some cities and months can have higher correlation between the index P and incidence of arbovirus disease that will not necessarily be expected in other periods or space. That is, although increases in the index P suggest a rise in the risk of mosquito-borne transmission, such an increase in risk of transmission is not necessarily linear. In Table 2, for instance, the index P increased from 2.23 to 2.24, that is 0.82%, between September and October of 2012. During that period the incidence of dengue increased by 79.4%. A year later, when hurricane Manuel hit Guerrero, the index P increased from 1.84 to 1.95 between September and October of 2013. This increase of 5.74% in the index P was reflected in a substantial increase in the incidence of dengue of 186.8%. The rise in dengue was not as high as the one we would have expected had the index P and risk transmission followed a linear relationship. There are many reasons for this finding. As mentioned earlier, the incidence of mosquito-borne diseases depends on more factors not considered in the index P such as population mobility, opportunities for the mosquito population to breed, deforestation, etc. Still, the index P provides a good tool to assess increased risk of mosquito-borne disease transmission.

## Discussion

Even though transmitted by the same vector, we showed that dengue, chikungunya and Zika can follow different spatial outbreak dynamics. Among all these diseases, dengue is the most widely spread in the country and its epidemiological spectrum remains a mix of epidemic, endemic, and hyperendemic areas. Over half of dengue cases are concentrated in about 65 municipalities in coastal, particularly by the Gulf, tropical areas and the Yucatán peninsula, all of which are important tourism and trade centres [9]. The annual economic impact of dengue in Mexico is estimated to be 130 million US dollars. Roughly 30% of these costs are in terms of direct medical costs and remaining in terms of patients’ economic costs [44]. Thus, it is paramount that timely surveillance tools are designed to help health authorities and scholars determine the seasons and areas most at risk of mosquito-borne diseases.

We also showed that the index P provides a reliable tool to estimate the transmission potential of mosquito-borne diseases. That is, the index P reveals quite well which areas are most at risk of transmission and crucially when. The index P also provides important insights into transmission during a wide range of climatic patterns and following extreme weather shocks including hurricanes. Although there are various alternative mosquito-borne potential transmission indices (such as water-associated diseases, and R0 mosquito-borne pathogens [11, 45]) these earlier indices rely on the complex interplay between social, economic, viral and entomological factors that are difficult to parameterise or model regularly and across broad regions [18]. It is rarely the case to have optimal mosquito populations or epidemiological data across the country and over time. Instead, the index P has the main advantage of relying exclusively on local humidity, temperature, and precipitation data, and a few vectors and human prior parameters already established in scientific literature. We showed that this analysis can be done with climate data that is usually readily available daily and of fine spatial scale. Timely information like this is vital to detect the highest viral transmission potential in each location and potential public health interventions to slow down the transmission of mosquito-borne viruses.

We acknowledge that our analysis has some limitations. Because of the simplicity of the index P, our study has not considered other potential important factors that could affect the transmission potential of mosquito-borne viruses such as existing public interventions to control mosquito population, socio-economic characteristics of the population, quality of housing conditions, among others. This limitation might explain why the index P has a lower correlation with mosquito-borne diseases in the north than in the south of the country. The north compared to the south is significantly wealthier, with better housing conditions, and subjected to distinct migration patterns. Thus, although the index P can be an important tool to promptly anticipate risks of transmission, effective surveillance systems are required to collect other vital epidemiological, ecological, entomological information. Still, the index P can serve as a baseline for the extent to which the climate alone contributes to risk of transmission. This is a powerful tool that can be used in future studies to measure the impact of interventions that seek to reduce mosquito population.

Future studies could also estimate the index P for much longer time periods than ours. This type of analysis could help assess to what extent climate change alone has affected the transmission risk of mosquito-borne diseases. Other studies could also use the index P and estimate for more recent periods how climatic variables alone affected the risks of transmission before and after population movement restrictions were implemented to contain the COVID-19 pandemic.

## Conclusions

Over the last fifty years, arboviral diseases have dramatically spread, in particular dengue which increased 30-fold and remains the most important and fastest-growing mosquito-borne viral disease worldwide. Mosquito-borne diseases can be reduced by adopting better surveillance tools for outbreak prediction, detection, and prompt controlling of mosquito populations. In this paper, we put into practice the recently proposed mosquito-borne viral suitability index P. This index estimates the transmission potential of mosquito-borne diseases such as dengue, chikungunya and Zika, identifying the areas and seasons most at risk.

Our analysis offered two important insights. First, our analysis revealed the index P to be strongly correlated with the incidence of dengue, its peaks during the year and spatial distribution within the country. Second, this correlation was also high enough for chikungunya and Zika to serve as an additional surveillance tool for these diseases in as vast a country as Mexico. Thus, our analysis suggests the index P can serve as an additional tool for surveillance systems in the country and in settings that have limited entomological information, epidemiological capacity, and exposed to rapidly changing climatic conditions. It is only with detailed analysis like this that policymakers and researchers can unravel the extent to which changing climate conditions affect the spread of mosquito-borne diseases and act promptly.

### Supplementary Information

**Additional file 1: Table S1.** Average monthly index P per meteorological station, aggregated at state level, during period 2000–2020.

## Data Availability

The datasets analysed during the current study and the replication code are available from the corresponding authors on reasonable request.

## Appendix

See Table 3, Figs. 13, 14 and 15

**Table 3:** Prior distribution used to estimate index P

Parameter	Notation	Mean	Stdev	Reference studies taken by Obolski et al. [11]
adult mosquito life-span	${1 \mathord{\left/ {\vphantom {1 {\mu_{(u,t)}^{v} }}} \right. \kern-\nulldelimiterspace} {\mu_{(u,t)}^{v} }}$	12 days	2	[24]; [26]
mosquito incubation period	${1 \mathord{\left/ {\vphantom {1 {\gamma_{(t)}^{v} }}} \right. \kern-\nulldelimiterspace} {\gamma_{(t)}^{v} }}$	7 days	2	[27]; [28]; [29]
mosquito biting rate	$a_{(u)}^{v}$	0.25/day	0.01	[30]; [25]
human life-span	1/*μ*^*h*^	71 years	3	–
human incubation period	1/*γ*^*h*^	5.8 days	1	[31]; [27]; [32]
human infections period	1/*σ*^*h*^	5.9 days	1	[31]; [27]; [32]
human to mosquito probability of transmission	$\phi^{h \Rightarrow v}$	0.5	0.01	–

Source: Obolski et al. [18]

## Abbreviations

MVSE: Mosquito-borne viral suitability estimator
IDW: Inverse distance weighted
HIP: Human incubation period
MLE: Mosquito life expectancy

## Footnotes

1 The R-package also informs users about the acceptance rate to assess whether the Bayesian inference reached convergence of the estimated Markov Chain Monte Carlo (MCMC) chain. For our case, the mean acceptance rate is 28.65% for the index P estimations across all the 188 automatic weather stations analysed during 2000–2020. In over 85.1% of the automatic stations, the acceptance rate reached the ideal 23% threshold or higher, suggesting that our study has a high acceptance rate of convergence.

2 Some automatic meteorological stations break down from time to time, during which it is not possible to estimate weather data for a few days during the year. For instance, for Mexico City, there are missing weather data from 2016–05-27 to 2016–11-28; for Acapulco from 2006–02-12 to 2006–09-21 and from 2007–07-12 to 2009–10-02; and for Cancún from 2005–10-22 to 2007–01-16 and from 2010–09-23 to 2011–12-18.

3 The index P depicted in Fig. 7 is estimated using 1,000 simulations for each day during 2000–2020. To generate each of these simulations the MVSE R package uses Eq. (3) which relies on eight parameters as priors. Then, for every year, we determine in which day the index P peaked for each of the simulations.

4 Some automatic meteorological stations sometimes did not report climate data for a few hours or days, presumably due to breakdowns. Thus, for the national analysis, we include automatic meteorological stations with: i) no more than 30% of missing values in a month (otherwise that month was discarded) and; ii) with valid months (according to condition i) for a whole year (otherwise, that year was excluded). This approach yields all the 188 automatic meteorological stations available. As a robustness check, we used other criteria to clean the weather data, and deal with missing values which yield nearly identical indices P, to the ones presented here.

5 The MVSE R package estimates the index P using climatic variables. This package applies a smoothing filter to these variables and standardises humidity and precipitation for numerical stability. We carried out this filter and standardisation using the same MVSE R package. In Table 2, we present all the climate variables without such a filter or standardisation, in raw units, merely to help appreciate the levels of these climatic variables in these two periods.
